# Olesoxime Inhibits Cardioplegia-Induced Ischemia/Reperfusion Injury. A Study in Langendorff-Perfused Rabbit Hearts

**DOI:** 10.3389/fphys.2017.00324

**Published:** 2017-05-19

**Authors:** Aida Salameh, Maren Keller, Ingo Dähnert, Stefan Dhein

**Affiliations:** ^1^Clinic for Pediatric Cardiology, Heart Centre, University of LeipzigLeipzig, Germany; ^2^Rudolf-Boehm-Institute for Pharmacology and Toxicology, University of LeipzigLeipzig, Germany

**Keywords:** cardioplegia, olesoxime, Langendorff heart preparation, cardioprotection, ATP

## Abstract

**Objective:** During cardioplegia, which is often used in cardiac surgery, the heart is subjected to global ischemia/reperfusion injury, which can result in a post-operative impairment of cardiac function. Mitochondria permeability transition pores (MPTP) play a key role in cardiomyocyte survival after ischemia/reperfusion injury. It was shown in clinical settings that blockers of MPTP like cyclosporine might have a positive influence on cardiac function after cardioplegic arrest. Olesoxime, which is a new drug with MPTP blocking activity, has been introduced as a neuroprotective therapeutic agent. This drug has not been investigated on a possible positive effect in ischemia/reperfusion injury in hearts. Therefore, the aim of our study was to investigate possible effects of olesoxime on cardiac recovery after cardioplegic arrest.

**Methods:** We evaluated 14 mature Chinchilla bastard rabbits of 1,500–2,000 g. Rabbit hearts were isolated and perfused with constant pressure according to Langendorff. After induction of cardioplegic arrest (30 ml 4°C cold Custodiol cardioplegia without and with 5 μmol/L olesoxime, *n* = 7 each) the hearts maintained arrested for 90-min. Thereafter, the hearts were re-perfused for 60 min. At the end of each experiment left ventricular samples were frozen in liquid nitrogen for ATP measurements. Furthermore, heart slices were embedded in paraffin for histological analysis. During the entire experiment hemodynamic and functional data such as left ventricular pressure (LVP), dp/dt(max) and (min), pressure rate product (PRP), coronary flow, pO_2_, and pCO_2_ were also assessed.

**Results:** Histological analysis revealed that despite the same ischemic burden for both groups markers of nitrosative and oxidative stress were significantly lower in the olesoxime group. Moreover, hearts of the olesoxime-group showed a significantly faster and better hemodynamic recovery during reperfusion. In addition, tissue ATP-levels were significantly higher in the olesoxime treated hearts.

**Conclusions:** Olesoxime significantly protected the cardiac muscle from ischemia/reperfusion injury.

## Introduction

During conventional cardiac surgery using heart-lung machine the heart is arrested by cardioplegic solutions. Depending on the type of operation cardiac arrest is maintained for up to 90 min or even longer. Although, cardioplegia and hypothermia provide certain protective effects regarding cardiac metabolic processes the heart is subjected to global ischemia/reperfusion injury. Therefore, heart protection during cardio-pulmonary bypass (CPB) is still a matter of debate. Experimental approaches range from improved management of cardiac cardioplegia, new cardioplegic solutions to improved flow profiles of the CPB (Salameh and Dhein, 2015). Ischemia and hypoxia during cardioplegic arrest result in respiratory chain failure and might finally initiate cell death (Luo et al., 2015). Hypoxic conditions during cardioplegia as well as reperfusion injury may result in reduced contractile function and promote arrhythmia, a situation in which catecholamine treatment is usually necessary with additional antiarrhythmic medication or electrical defibrillation and which has a negative impact on patient outcome (Murnaghan, 1975; Casthely et al., 1994).

The key factor during insufficient perfusion of cardiac tissue is the breakdown of mitochondrial activity i.e., oxidative phosphorylation. As ATP (adenosine triphosphate) cannot be stored intracellularly the breakdown of ATP has serious consequences for function and survival of cardiomyocytes. Due to hypoxic conditions cardiac metabolic processes have to switch from aerobic to anaerobic glycolysis with the result of insufficient ATP-production and intracellular acidosis, which then in turn will lead to an activation of the Na^+^/H^+^- and the Na^+^/Ca^2+^-exchanger (Clanachan, 2006). The activation of ion-exchangers and the failure of membrane pumps (which are also ATP-dependent) cause intracellular sodium and calcium overload with loss of resting potential and cellular swelling. Calcium overload and -during reperfusion- increased levels of reactive oxygen or nitrogen species might provoke prolonged opening of mitochondrial permeability transition pores (MPTPs) resulting in mitochondrial swelling and rupture, cytochrome c release, cessation of ATP synthesis, AIF (apoptosis inducing factor) liberation and induction of cell apoptosis or necrosis (Borutaite et al., 2013; Kwong and Molkentin, 2015).

Hence, prolonged opening of MPTPs has negative effects on heart function (Kwong and Molkentin, 2015) and it is conceivable that blockade of MPTPs might prevent from mitochondrial damage and might preserve cardiac contractility.

Olesoxime is a new cholesterol-like drug developed for the therapy of neurodegenerative diseases like amyotrophic lateral sclerosis or spinal muscular atrophy (Bordet et al., 2007, 2010). It seems to be beneficial especially for the therapy of spinal muscular atrophy and at the moment phase II clinical trials are ongoing for the application of olesoxime in this indication (Calder et al., 2016). The interesting feature of this drug is its ability to block MPTPs, thereby preventing from mitochondrial swelling which often precedes cell death (Lemasters et al., 2009).

Since olesoxime has not yet been tested in cardiac arrest we wanted to evaluate if this drug might be beneficial for recovery of heart function after cardioplegia. For that purpose rabbit hearts were arrested for 90 min without or with olesoxime followed by 60 min of reperfusion, during which hemodynamic and electrophysiological parameters were assessed. ATP-measurements and histological evaluation of apoptotic markers, as well as markers for oxidative and nitrosative stress were carried out at the end of the experiment.

## Methods

All experiments were performed in accordance with the ethical rules of the Council for International Organization of Medical Science and the German/European laws for animal welfare. The study was approved by our institutional ethical committee for animal welfare and the regional council of Leipzig named “Landesdirektion Sachsen” (reference number T21/16). The investigation conforms to the Directive 2010/63/EU of the European Parliament as well as to the Guide for the Care and Use of Laboratory Animals published by the US National Institutes of Health (NIH Publication No. 85-23, revised 1996).

For our experiments we used mature female and male Chinchilla rabbits weighting 1,500–2,000 g. Preparation of the hearts and perfusion according to the Langendorff-technique was performed as described earlier (Salameh et al., 2015b).

In brief, animals were anesthetized with medetomidine (0.2 mg/kg body weight) and ketamine (20 mg/kg body weight). Heparine (500 IU/kg body weight) was administered intravenously. After induction of narcosis the rabbits were exsanguinated, the thorax was opened and the ascending aorta as well as the pulmonary artery were cannulated (1 min until aortic cannulation). Vena cava superior and inferior as well as the pulmonary veins were ligated separately. The hearts were quickly removed and connected to the Langendorff apparatus. Coronary arteries were retrogradly perfused at constant pressure of 75 cmH_2_O with Tyrode's solution (containing (in mmol/L) Na^+^ 161, K^+^ 5.36; Ca^2+^ 1.8, Mg^2+^ 1.05; Cl^−^ 148, ${\text{HCO}}_{3}^{-}$ 23.8, ${\text{PO}}_{4}^{3-}$ 0.42 and glucose 11.1; gassed with 5% CO_2_ and 95% O_2_) and coronary flow (CF ml/min) was measured via the pulmonary artery. Left atrium was opened and the pressure balloon was introduced into the left ventricle and filled with water to achieve an end-diastolic pressure of 8.3 ± 0.18 mmHg. The pressure catheter was connected to a transducer and a two-channel bridge amplifier (both from Hugo Sachs Elektronik, March-Hugstetten, Germany). Pressure traces were continuously recorded (trace recorder: Recomed, Hellige, Freiburg, Germany) to determine left ventricular systolic pressure (LVP), left ventricular end-diastolic pressure (EDP), dp/dt (max), and (min), basic cycle length (BCL) and heart rate (HR).

Four polyester plates with 64 electrodes each (i.e., 256 AgCl electrodes) were attached onto the surface of the heart in an elastic manner to allow a resonation of the electrode blocks with the spontaneously beating hearts. The electrodes were connected to a 256 channel mapping system HAL4 (temporal resolution: 4 kHz/channel; amplitude resolution: 0.04 mV) to measure surface ECGs from the right and left ventricular free wall and the anterior and posterior wall. Activation recovery interval (ARI), total activation time (TAT), PQ-interval, QRS width, corrected QT interval (cQT), and vector field similarity were assessed according to Dhein et al. (1993).

Additionally, via the pulmonary cannula pO_2_ (partial oxygen pressure in mmHg), pCO_2_ (partial carbon dioxide pressure in mmHg) and coronary flow (measured volumetrically) (ml/min) were assessed during the entire experiment. Oxygen partial pressure was measured in Tyrode's solution (“arterial”) and in the outflow of pulmonary cannula (“venous”) and the “arterio-venous” difference was calculated which is a measure for the oxygen extraction of the beating heart.

### Experimental protocol

Fourteen rabbits were investigated and divided into two groups (each group consisted of 4 female and 3 male rabbits): cardioplegia group without olesoxime (CP-) and CP-group with 5 μmol/L olesoxime (CP+). The concentration of 5 μmol/L olesoxime was chosen according to Bordet et al. (2007, 2010).

After an equilibration period of 45 min baseline hemodynamic parameters and epicardial electrograms were recorded as well as arterial and venous pO_2_ and pCO_2_. Afterwards, cardioplegia (without olesoxime or with 5 μmol/L olesoxime) was initiated by coronary injection of 30 ml 4°C cold Custodiol® (Dr. Franz Köhler Chemie, Alsbach-Hähnlein, Germany) containing (in mmol/L): NaCl 15, KCl 9, MgCl_2_ 4, CaCl_2_ 0.015, histidinehydrochloride 18, L-histidine 180, tryptophan 2, mannitol 30, and ketoglutarate/glutamic acid 1. A stock solution of 5,000 μmol/L olesoxime was prepared in DMSO (dimethyl sulfoxide), diluted in Custodiol® and added to the cardioplegia solution to achieve a final concentration of 5 μmol/L. The final concentration of DMSO within the cardioplegia solution was 1:1000. The surface temperature of the heart was measured by a thermometer placed on the cardiac surface. The temperature was maintained at 18°C by over-spilling the heart's surface with Tyrode's solution at 0.5 ml/h.

After 90 min of cardioplegia, reperfusion with Tyrode's solution was initiated and the above described parameters were acquired at 5, 10, 20 30, and 60 min.

Subsequently, the hearts were quickly removed from the Langendorff-apparatus and samples from the left ventricular apex were shock frozen in liquid nitrogen for ATP analysis. For histological analysis transverse slices of the heart were fixed in 4% neutral buffered formalin and embedded in paraffin.

### Histology

Histological evaluation of the ventricular probes was carried out according to Salameh et al. (2015b), in order to analyze the presence of the pro-apoptotic factor AIF (apoptosis inducing factor), PAR (poly-ADP-ribose)-formation, HIF1α hypoxia induced factor 1α-translocation and cC3 (cleaved caspase 3)-levels as well as nitrotyrosine formation and HSP60 (heat shock protein 60)-levels. Specimens embedded in paraffin were cut in 2 μm slices, de-paraffinized and treated with 0.3% Triton-X 100 (for nitrotyrosine-staining) or cooked in 0.01 mol/L citrate buffer (*pH* = 6) (for AIF-, HIF1α-, PAR-, cC3-, and HSP60-staining). Thereafter, histological specimen were blocked with BSA (bovine serum albumin) and treated with either mouse monoclonal anti-nitrosine primary antibody (1:50, Merck-Millipore, Darmstadt, Germany), mouse monoclonal anti-PAR antibody (1:600, Bio-Rad, Munich), mouse anti-AIF primary antibody (1:50, Santa Cruz, Heidelberg, Germany), with mouse monoclonal anti-HIF1α primary antibody (1:50, Thermo Scientific, Dreieich, Germany), with mouse monoclonal HSP60 primary antibody (1:250, Abcam, Cambridge, UK) or with rabbit anti-cC3 primary antibody (1:300, New England Biolabs, Frankfurt, Germany) over-night at 4°C.

After several washing steps, HRP-labeled secondary goat anti-mouse or goat anti-rabbit antibodies (1:200, Sigma-Aldrich, Taufkirchen, Germany) were applied for 2 h. Subsequently, specimens were washed again and incubated with the red chromogen AEC (3-amino-9-ethylcarbazole, Dako, Hamburg, Germany) according to the manufacturer's instruction. Nuclei of cardiomyocytes were counter-stained with haemalum.

The slides were investigated at 400 × magnification using a Zeiss Axiolab microscope (Zeiss, Jena, Germany) and photographs were randomly taken by a blinded investigator. The following heart regions were analyzed: for the right ventricle the mid-myocardial region of the right ventricular free wall (RV) was studied and for the left ventricle (LV) three regions of the free LV wall were assessed separately: epicardium, mid-myocardium and endocardium. These regions can be discriminated microscopically by the direction of the fibers. Endocardium makes about the inner 1/4 of the wall, epicardium the outer 1/4, and mid-myocardium the remaining 2/4 in the middle of the wall (Streeter et al., 1969).

Each region was analyzed separately and at least 50 cells per region were counted and the ratio of positive cells (red nuclei or red cytoplasm) was evaluated in relation to the total number of counted cells. Accordingly, 200 cells per heart and 1,400 cells per experimental group were investigated.

### HPLC analysis of ATP

Left ventricular specimen were homogenized at 4°C with 0.4 mmol/L perchloric acid and precipitated with KOH. Thereafter, the samples were centrifuged for 10 min and 20 μl of the supernatant was injected twice onto a pre-equilibrated RP18 column (LiChroCART, Merck, Darmstadt, Germany) as previously published (Salameh et al., 2015a). For ATP, ADP AMP, and adenosine detection a HPLC-apparatus from Knauer (Berlin, Germany) and an UV-detector (PDA Detector 2800, Knauer, Berlin, Germany) were used. Peaks were measured at 259 nm, standard curves were generated with 4 concentrations of ATP, ADP, AMP, and adenosine respectively (25–12.5–6.25–3.125 μg/ml) and measured together with the ventricular samples.

### Statistical analysis

All results are presented as mean value and standard error of mean (SEM) of *n* = 7 experiments. Normal distribution of the data was tested with Shapiro-Wilk's test.

Hemodynamic data were evaluated with repeated measurements ANOVA followed by Students *t*-test with Bonferroni correction for multiple measurements to detect statistical significance at a level of *p* < 0.05. ATP measurements were tested for statistical significance using ANOVA followed by Students *t*-test, and the non-parametric Kruskal-Wallis test was performed to analyse the histological data (*p* < 0.05).

Prevalence of ventricular fibrillation in both experimental groups was tested using Fischer's exact test.

For the statistical analyses Systat for Windows, version 13 (Systat Inc., Evanston, IL, USA) was used.

## Results

Two groups of rabbits were evaluated: CP- (body weight 1,778 ± 26 g, heart weight 7.5 ± 0.14 g, *n* = 7) and CP+ with 5 μmol/L olesoxime administered during cardioplegia (body weight 1,862 ± 15 g, heart weight 7.2 ± 0.1, *n* = 7). Both groups did not significantly differ in body and heart weight.

### Physiological, hemodynamic, and electrophysiological parameters and tissue ATP-content

At the end of the equilibration period baseline values of hemodynamic and electrophysiological parameters were not significantly different in both experimental groups (see Table 1).

**Table 1:** **Physiological, biochemical and hemodynamic parameters**.

	**Baseline**		**Reperfusion 5 min**		**Reperfusion 10 min**		**Reperfusion 20 min**		**Reperfusion 30 min**		**Reperfusion 60 min**	
	**CP−**	**CP+**	**CP−**	**CP+**	**CP−**	**CP+**	**CP−**	**CP+**	**CP−**	**CP+**	**CP−**	**CP+**
ARI (ms)	143 ± 4.29	137 ± 6.0	158 ± 5.4	132 ± 6.5^*^	152 ± 4.1	144 ± 4.2	150 ± 5.4	140 ± 5.1	148 ± 5.3	136 ± 4.8	144 ± 5.5	143 ± 6.1
TAT (ms)	16.29 ± 1.81	17.13 ± 0.79	20.70 ± 1.32	18.00 ± 1.10	21.70 ± 2.05	17.79 ± 1.53^*^	20.50 ± 1.09	16.25 ± 1.83^*^	21.00 ± 1.84	16.05 ± 0.98^*^	20.92 ± 2.10	16.05 ± 0.73^*^
cQT	219 ± 6.5	217 ± 7.7	234 ± 8.7	241 ± 13.0	250 ± 7.8	231 ± 5.7^*^	226 ± 6.1	213 ± 6.2	223 ± 5.6	217 ± 5.7	230 ± 6.2	220 ± 5.2
PQ (ms)	63 ± 2.0	58 ± 2.2	63 ± 4.0	59 ± 2.1	64 ± 3.7	61 ± 2.0	64 ± 2.5	60 ± 2.4	65 ± 2.1	63 ± 2.4	61 ± 2.7	65 ± 2.8
QRS (ms)	26 ± 0.71	26 ± 0.61	25 ± 0.46	25 ± 0.70	27 ± 0.97	24 ± 0.35	25 ± 0.43	24 ± 0.18	24 ± 0.47	24 ± 0.49	26 ± 0.75	24 ± 0.25
BCL (ms)	408 ± 8.9	396 ± 9.9	491 ± 48.7	312 ± 10.7^*^	377 ± 10.6	382 ± 9.6	435 ± 11.2	447 ± 14.2	447 ± 20.7	388 ± 13.4	390 ± 12.2	421 ± 16.4
HR (min^−1^)	155 ± 3.5	155 ± 3.3	167 ± 4.0	192 ± 6.3^*^	163 ± 4.6	161 ± 3.9	141 ± 3.7	140 ± 4.7	141 ± 5.0	156 ± 5.1	158 ± 4.4	151 ± 5.1
CF/heart weight (ml/min/g)	3.18 ± 0.11	3.20 ± 0.08	3.08 ± 0.16	3.31 ± 0.21	2.86 ± 0.13	3.17 ± 0.13	2.52 ± 0.11	3.03 ± 0.12	2.57 ± 0.12	2.98 ± 0.13	2.40 ± 0.06	2.86 ± 0.14
Lactate (mmol/L)	0 ± 0	0 ± 0	0.07 ± 0.02	0.05 ± 0.03	0.02 ± 0.01	0 ± 0	0.01 ± 0.02	0 ± 0	0.01 ± 0.02	0 ± 0	0.02 ± 0.02	0.01 ± 0.01
pO_2_art-ven (mmHg)	347 ± 9.2	379 ± 8.2	316 ± 13.2	369 ± 9.3^*^	311 ± 10.9	367 ± 8.5^*^	364 ± 8.6	415 ± 8.5^*^	362 ± 11.4	433 ± 8.1^*^	368 ± 11	441 ± 9.1^*^
pCO_2_ (mmHg)	41 ± 0.5	39 ± 0.5	41 ± 0.8	40 ± 1.0	40 ± 0.7	38 ± 0.7	39 ± 1.0	38 ± 0.7	40 ± 0.9	39 ± 0.6	40 ± 0.8	39 ± 0.7
ATP content μg/g											56 ± 4.6	78 ± 7.7^*^

*ARI, activation recovery interval; TAT, total activation time; cQT, corrected QT interval; PQ, PQ interval; QRS, QRS complex; BCL, basic cycle length; HR, heart rate; CF, coronary flow; pO_2_art-ven difference of arterio-venous partial oxygen pressure, pCO_2_ partial carbon dioxide pressure, ole olesoxime*.

*CP− cardioplegia, CP+ cardioplegia+olesoxime*.

* *Significant differences CP− vs. CP+ (p < 0.05)*.

During the reperfusion period pCO_2_ was not significantly different between CP- and CP+. However, during reperfusion arterio-venous pO_2_ difference was higher in CP+ compared to CP- (Table 1) i.e., cardiac oxygen extraction of the hearts treated with olesoxime during CP was higher compared to CP without olesoxime. Additionally, coronary flow (related to heart weight) was slightly (but not significantly) increased in the olesoxime group during recovery (Table 1). Moreover, lactate concentration in the coronary effluent was low and not significantly different between CP- and CP+ (Table 1). Heart rate was found to be significantly lower at 5 min reperfusion in CP- hearts (Table 1). At the same time CF/PRP was elevated in these hearts (**Figure 2A**).

**Figure 1:**
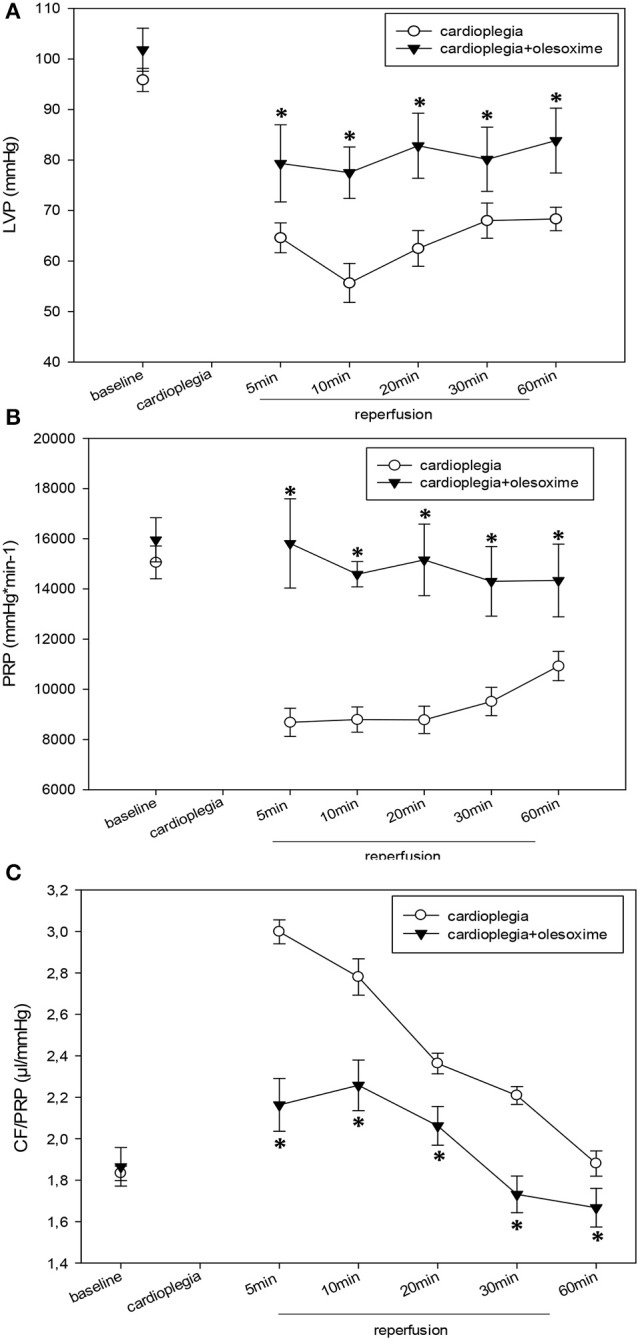
**(A)**. Left ventricular pressure (LVP) in mmHg before (baseline) and during recovery from a 90 min period of cardioplegia. Cardioplegia was performed either without (white circles, *n* = 7) or with addition of 5 μmol/L olesoxime (black triangles, *n* = 7). All data are given as means ± SEM. Significant differences (*p* < 0.05) between CP− and CP+ are indicated by asterisks (^*^). **(B)** End-diastolic pressure (EDP) in mmHg before (baseline) and during recovery from a 90 min period of cardioplegia. Cardioplegia was performed either without (white circles, *n* = 7) or with addition of 5 μmol/L olesoxime (black triangles, *n* = 7). All data are given as means ± SEM. Significant differences (*p* < 0.05) between CP− and CP+ are indicated by asterisks (^*^). **(C)** Pressure-rate-product (PRP) in mmHg^*^min^−1^ before (baseline) and during recovery from a 90 min period of cardioplegia. Cardioplegia was performed either without (white circles, *n* = 7) or with addition of 5 μmol/L olesoxime (black triangles, *n* = 7). All data are given as means ± SEM. Significant differences (*p* < 0.05) between CP− and CP+ are indicated by asterisks (^*^).

**Figure 2:**
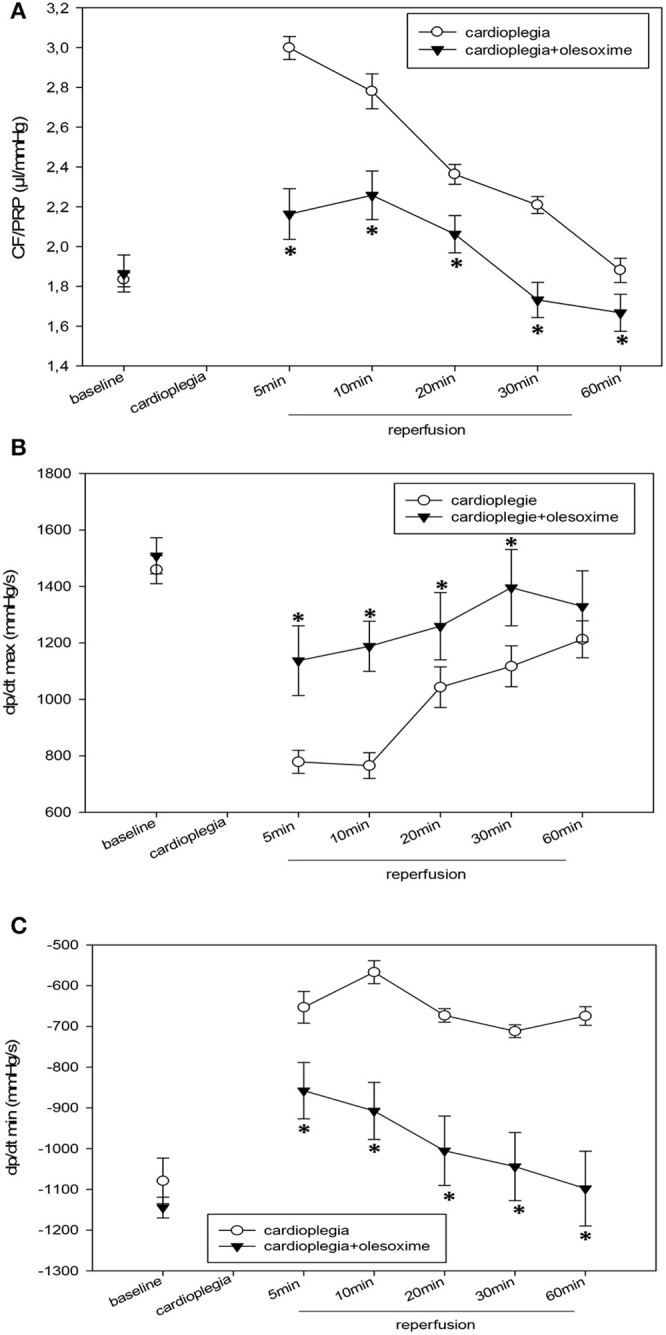
**(A)** Ratio of coronary flow and pressure-rate-product (CF/PRP) in μl/mmHg before (baseline) and during recovery from a 90 min period of cardioplegia. Cardioplegia was performed either without (white circles, *n* = 7) or with addition of 5 μmol/L olesoxime (black triangles, *n* = 7). All data are given as means ± SEM. Significant differences (*p* < 0.05) between CP− and CP+ are indicated by asterisks (^*^). **(B)** Maximum dp/dt (dp/dt max) in mmHg/s before (baseline) and during recovery from a 90 min period of cardioplegia. Cardioplegia was performed either without (white circles, *n* = 7) or with addition of 5 μmol/L olesoxime (black triangles, *n* = 7). All data are given as means ± SEM. Significant differences (*p* < 0.05) between CP− and CP+ are indicated by asterisks (^*^). **(C)** Minimum dp/dt (dp/dt min) in mmHg/s before (baseline) and during recovery from a 90 min period of cardioplegia. Cardioplegia was performed either without (white circles, *n* = 7) or with addition of 5 μmol/L olesoxime (black triangles, *n* = 7). All data are given as means ± SEM. Significant differences (*p* < 0.05) between CP− and CP+ are indicated by asterisks (^*^).

Analysis of hemodynamic parameters during reperfusion revealed that cardioplegia itself induces significant decreases in LVP, PRP (pressure-rate product) and dp/dt (max) and (min) compared to baseline values. EDP was significantly increased in the CP- group and only slightly elevated in CP+. Administration of olesoxime during cardioplegia resulted in a significantly improved hemodynamic outcome of the hearts: i.e., inotropy and lusitropy was significantly better in the CP+ group compared to CP- (Figures 1, 2). Indeed, hemodynamics of olesoxime treated hearts nearly reached baseline levels at the end of reperfusion. In addition, we found that CF/PRP ratio was significantly elevated in the CP- group shortly after reperfusion followed by a decline to the baseline level. In contrast, this CP-induced relative flow increase (μl/mmHg) was significantly reduced by olesoxime (Figure 2A).

Analysis of electrophysiological data revealed a slight prolongation of ARI and cQT intervals during early reperfusion in both groups (Table 1). Interestingly, TAT was prolonged only in the CP- but not in the CP+ group during reperfusion (Table 1).

Moreover, 3 out of 7 CP- hearts exhibited transient ventricular fibrillation within the first 2–3 min after reperfusion whereas none of the hearts in the CP+ group showed ventricular fibrillation. However, this increased prevalence of ventricular fibrillation in CP- failed to be statistically significant (*p* = 0.192). Ventricular fibrillation could be mechanically terminated. Other ECG parameters like PQ-interval, QRS width, BCL (which reflects heart rate) and beat-to-beat vector similarity were not different between both experimental groups (Table 1).

ATP-measurements revealed significant higher tissue ATP-levels in the CP+ compared to CP- (Table 1).

### Immunohistology

**Figure 3:**
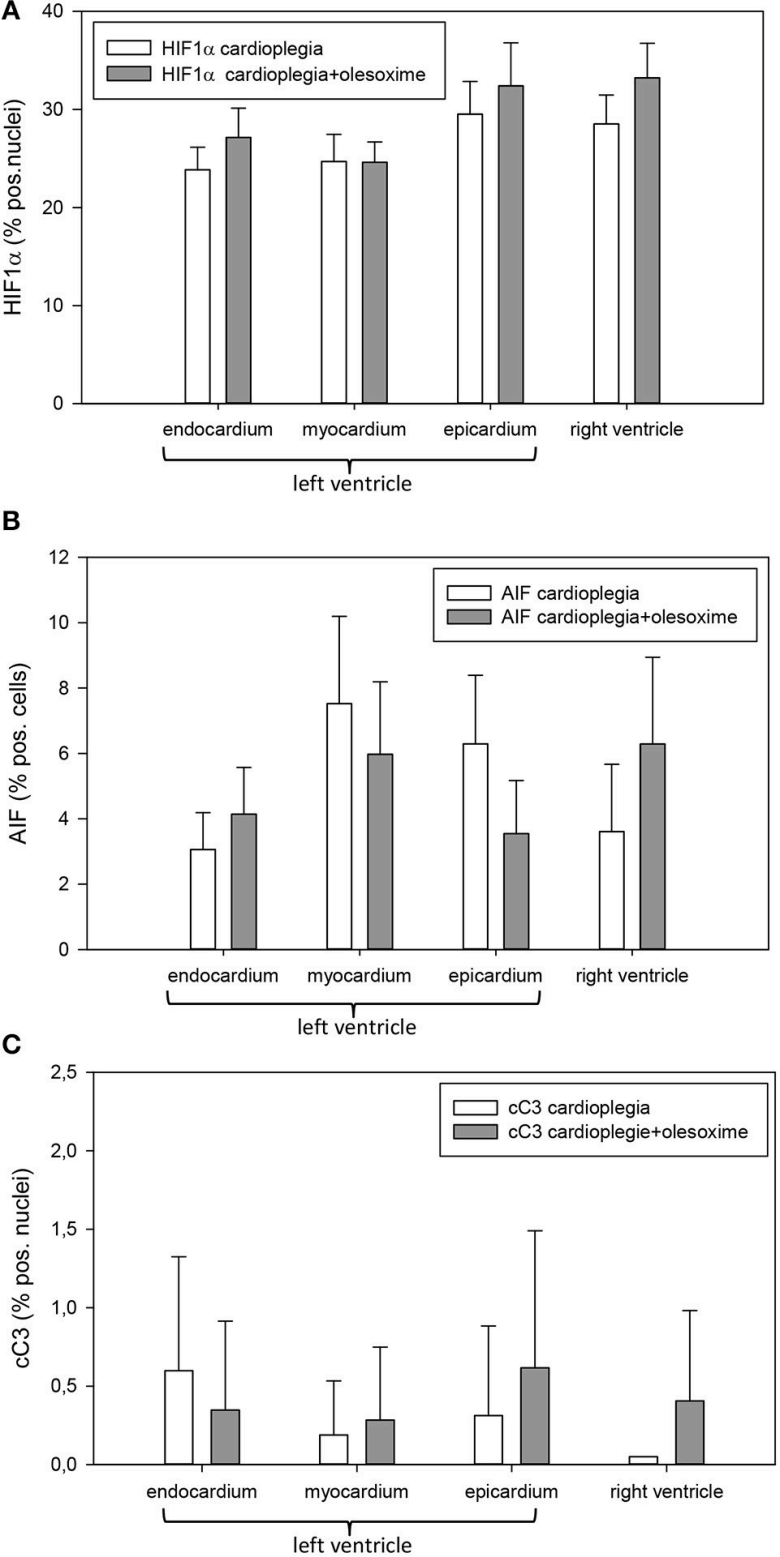
**(A)** Staining and quantification of HIF1α (hypoxia-inducible factor 1α) translocation. Bar graphs depict the percentage of nuclei positively stained for HIF1α in specimens from left ventricular epicardium, myocardium, and endocardium and right ventricle after 90 min of cardioplegia followed by 60 min of recovery. Both groups cardioplegia without olesoxime (*n* = 7) and cardioplegia with 5 mmol/L olesoxime (*n* = 7) did not differ significantly. All data are given as means ± SEM. **(B)** Staining and quantification of AIF (apoptosis-inducing factor). Bar graphs depict the percentage of cells positively stained for AIF in specimens from left ventricular epicardium, myocardium, and endocardium and right ventricle after 90 min of cardioplegia followed by 60 min of recovery. Both groups cardioplegia without olesoxime (*n* = 7) and cardioplegia with 5 mmol/L olesoxime (*n* = 7) did not differ significantly. All data are given as means ± SEM. **(C)** Staining and quantification of cC3 (cleaved caspase 3) translocation. Bar graphs depict the percentage of nuclei positively stained for cC3 in specimens from left ventricular epicardium, myocardium and endocardium and right ventricle after 90 min of cardioplegia followed by 60 min of recovery. Both groups cardioplegia without olesoxime (*n* = 7) and cardioplegia with 5 mmol/L olesoxime (*n* = 7) did not differ significantly. All data are given as means ± SEM.

**Figure 4:**
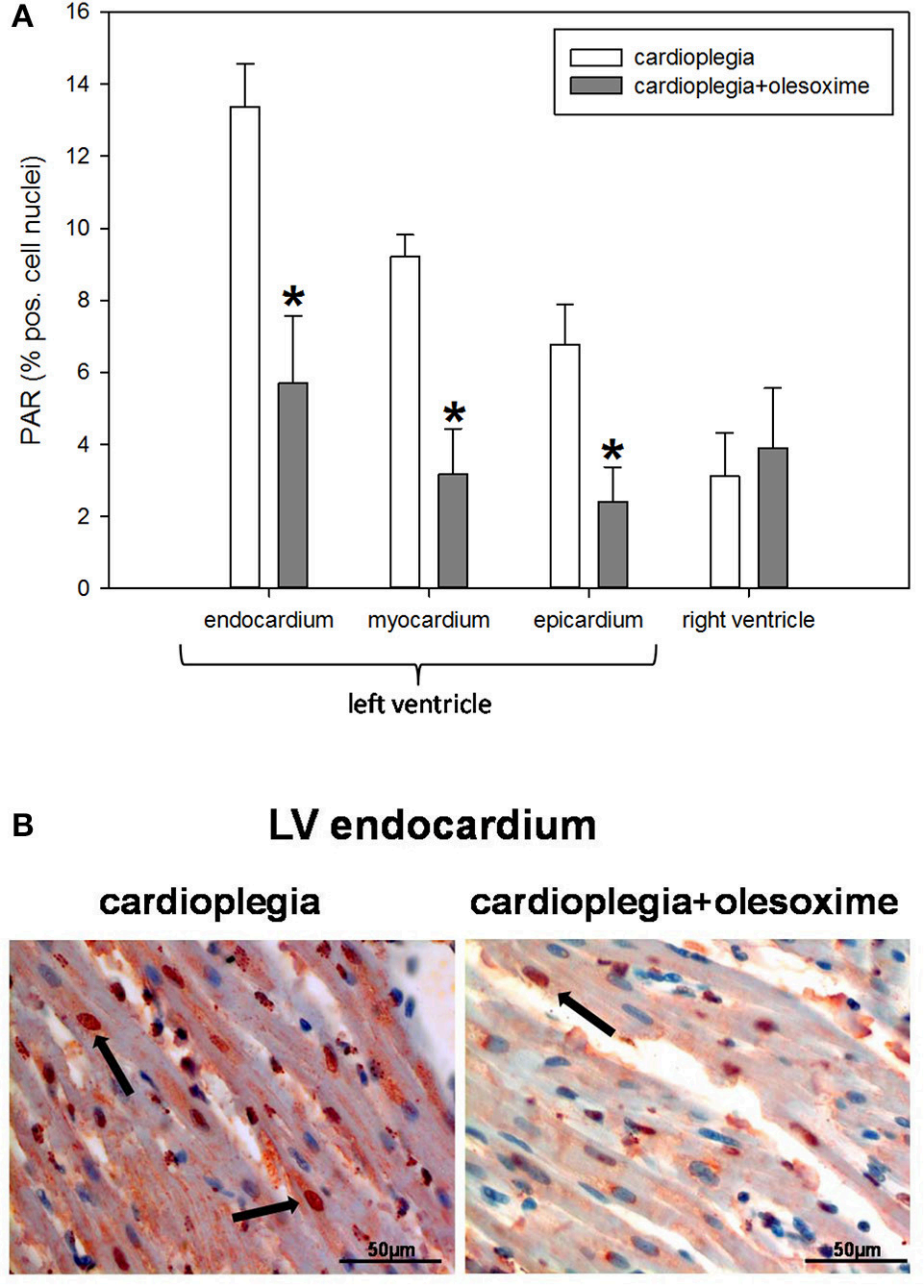
**(A)** Staining and quantification of nuclear PAR (poly-ADP-ribose) expression. Bar graphs depict percentage of nuclei positively stained for PAR in specimens from left ventricular epicardium, myocardium, and endocardium and right ventricle after 90 min of cardioplegia followed by 60 min of recovery. All data are given as means ± SEM. Significant differences (*p* < 0.05) between CP− and CP+ are indicated by asterisks (^*^). **(B)** Original PAR-staining of left ventricular (LV) endocardium. Arrows indicate cell nuclei positive for PAR (stained in red).

**Figure 5:**
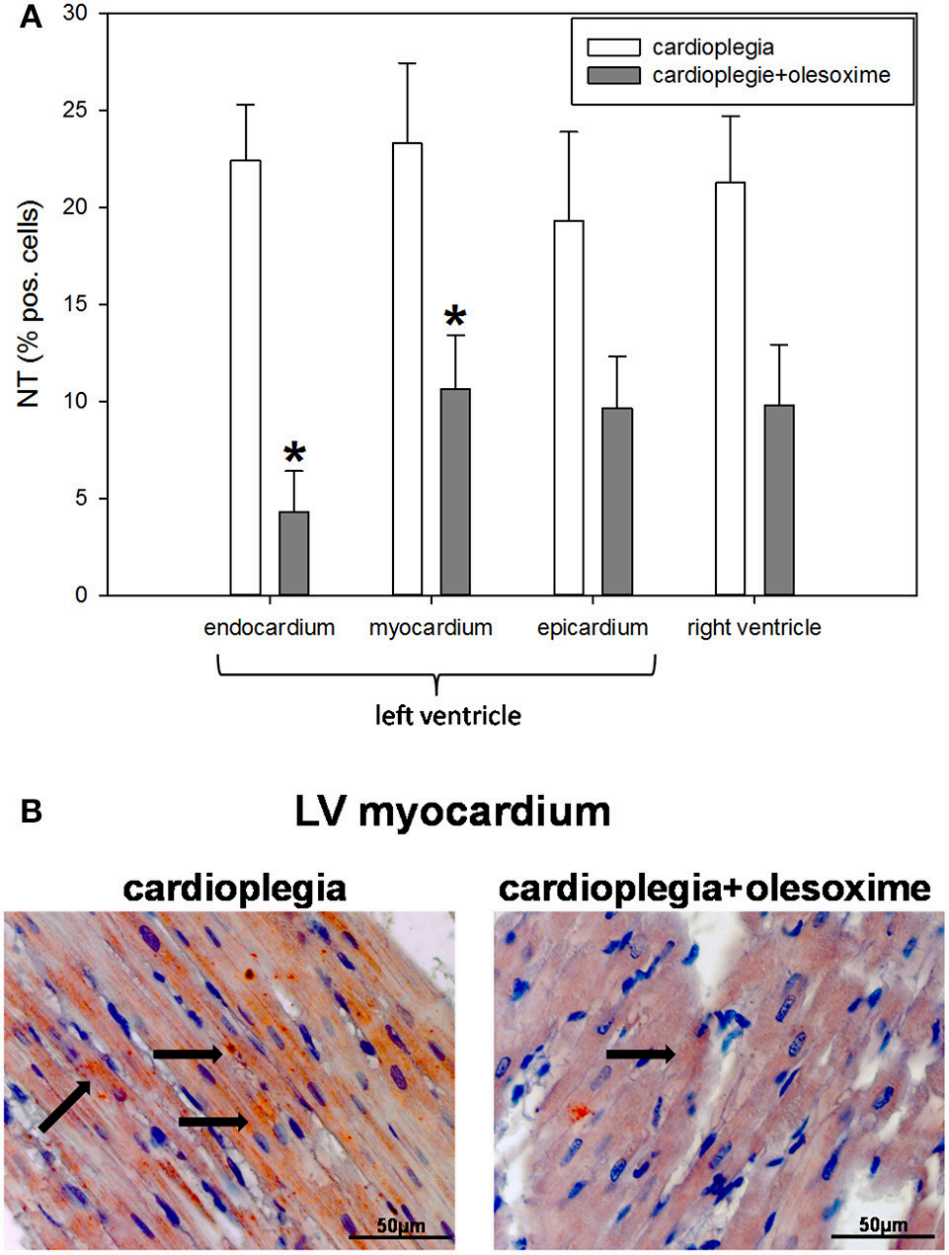
**(A)** Staining and quantification of nuclear NT (nitrotyrosine) expression. Bar graphs depict percentage of cells positively stained for NT in specimens from left ventricular epicardium, myocardium, and endocardium and right ventricle after 90 min of cardioplegia followed by 60 min of recovery. All data are given as means ± SEM. Significant differences (*p* < 0.05) between CP− and CP+ are indicated by asterisks (^*^). **(B)** Original NT-staining of left ventricular (LV) myocardium. Arrows indicate cells positive for NT (stained in red).

**Figure 6:**
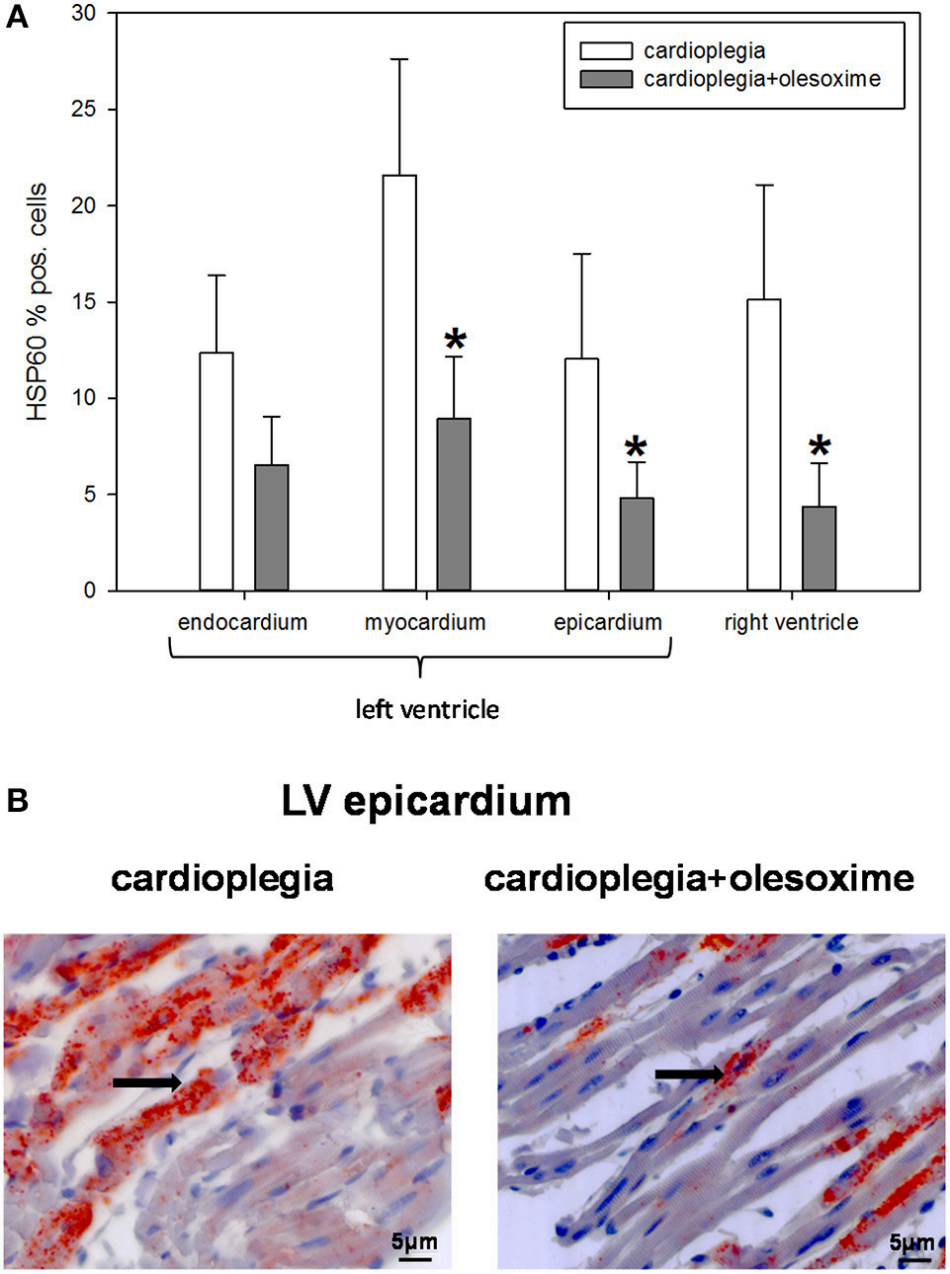
**(A)** Staining and quantification of HSP60 (heat shock protein 60) expression. Bar graphs depict percentage of cells positively stained for HSP60 in specimens from left ventricular epicardium, myocardium and endocardium and right ventricle after 90 min of cardioplegia followed by 60 min of recovery. All data are given as means ± SEM. Significant differences (*p* < 0.05) between CP− and CP+ are indicated by asterisks (^*^). **(B)** Original HSP60-staining of left ventricular (LV) epicardium. Arrows indicate cells positive for HSP60 (stained in red).

Histological analysis of HIF1α, AIF and cC3 showed no significant differences between the two experimental groups nor between the 4 different heart areas which were assessed (RV, LV epicardium, myocardium and endocardium; Figures 3A–C). Evaluation of NT and PAR however, revealed that olesoxime treatment significantly reduced elevated NT and PAR during cardioplegia. This effect was particularly evident in specimen of the left ventricle (Figures 4, 5). Additionally, expression of HSP60, a mitochondrial chaperonin, was also significantly reduced after olesoxime treatment during cardioplegia (Figure 6).

## Discussion

Our experimental data revealed that olesoxime significantly improved cardiac outcome after cardioplegia: left ventricular pressure, dp/dt (max) and (min), PRP -a measure for cardiac workload-, coronary flow, and ATP-levels were significantly higher in the olesoxime treated hearts. In the reperfusion phase after cardioplegia a slight prolongation of TAT occurred in the CP- group, which was accompanied by an enhanced incidence of ventricular fibrillation. Prolongation of TAT means a longer timespan for ventricular activation and thus slower ventricular conduction velocity. Slowing of conduction however is a well-known pro-arrhythmic factor. In contrast, olesoxime treated hearts did not exhibit prolonged TAT and did not develop ventricular fibrillation after cardioplegia. Moreover, cessation of ATP-synthesis due to cardioplegic arrest is known to result in failure of Na^+^/K^+^-ATPase, Na^+^/H^+^ exchanger, and Na^+^/Ca^2+^ exchanger which will lead to intracellular Na^+^-overload, the latter causing reduced conduction velocity as became apparent from TAT prolongation in the CP- group. In addition, the increase in relative CF, i.e., CF/PRP (μl/mmHg) after reperfusion indicates O_2_-deficit and consecutive metabolic autoregulation induced by the degradation of ATP.

Assuming that olesoxime inhibits MPTPs would mean that ATP-synthesis is maintained (Martin et al., 2011) and, thus the activation of metabolic autoregulation (i.e., the increase in CF/PRP) should be lower in the olesoxime group, which indeed is indicated by our data. Moreover, we assume that the better recovery of the olesoxime-treated hearts with higher ATP-levels should result in lower adenosine release, as indicated by a significantly lower CF/PRP ratio (Figure 2A). Since adenosine slows heart rate, the heart rate at early recovery in olesoxime hearts then should be higher, in good correspondence to our data.

Histological analysis revealed a significantly reduced formation of PAR and NT in the olesoxime treated hearts after cardioplegia. Moreover, HSP60, a mitochondrial chaperonine indicative for stress response was significantly lower in the olesoxime group.

Olesoxime a drug with neuroprotective properties is currently tested in clinical studies and seems to be useful in the therapy of spinal muscular atrophy.

Animal studies demonstrated that neuronal/cerebral ROS (reactive oxygen species) and RNS (reactive nitrogen species) production was diminished as was apoptosis after pretreatment with olesoxime (Martin et al., 2011; Ma et al., 2014). The mechanism behind these positive effects seems to be the inhibition of mitochondrial permeability transition by blockade of MPTPs. However, until now olesoxime has only been tested in neurological diseases. To our knowledge our study on the effect of olesoxime during cardioplegia is the first one showing that this drug might also be beneficial for preservation of cardiac function after cardioplegic arrest. Our data seem to support this hypothesis: although both experimental groups (CP- and CP+) had the same “ischemic burden,” which was demonstrated by the same amount of HIF1α nuclear translocation, hearts treated with olesoxime had a significantly better outcome regarding hemodynamic parameters. The reason may be an improved mitochondrial function because of MPTP-blockade by olesoxime with preservation of the respiratory chain. The finding of a reduction of elevated mitochondrial HSP60 after cardioplegia would also be in favor of better preserved mitochondria. In line with this theory Toga et al. (2007) could demonstrate in a rat model of myocardial infarction that HSP60 and mitochondrial oxygen consumption correlated inversely i.e., the higher HSP60 content the lower was the oxygen consumption rate.

Mitochondria play a key role in ischemia/reperfusion injury of the heart and are also one of the main producers of ROS and RNS during ischemia and reperfusion injury (Akopova et al., 2016; Madungwe et al., 2016). We could demonstrate that RNS production, which was measured indirectly by the formation of the more stable nitrotyrosine product (Wang et al., 2016) was significantly reduced in the olesoxime group. RNS like peroxynitrite induce DNA strand breaks, which subsequently activate poly-ADP polymerase (PARP) leading to enhanced PAR formation. PARP activation is on one hand an important process regarding cell survival as DNA damages are repaired but on the other hand a highly ATP consuming process, which lead to a further reduction in energy rich phosphates and might finally end in cell apoptosis or necrosis (Gerö et al., 2014). In our study PAR formation in specimen of the left ventricle was significantly reduced by half in the olesoxime group, indicating less PARP activation, which also might have contributed to the higher ATP-levels determined in the olesoxime treated hearts. Other pharmacological inhibitors of MPTP like cyclosporine A are known to be protective in models of neuronal ischemia and reperfusion injury (Fakharnia et al., 2016). Likewise, it was shown that inhibition of MPTPs by cyclosporine A was also effective in preventing kidney, liver and lung damage after cardiac resuscitation (Cour et al., 2014). Moreover, our working group recently demonstrated positive effects of cyclosporine A treatment on cardioplegic rabbit hearts (Pritzwald-Stegmann et al., 2011). However, in clinical trials the effect of cyclosporine A was limited: in a study with patients undergoing coronary artery bypass grafting a positive effect was only seen in high risk patients with longer bypass times. In a recently published study on cyclosporine A application in patients with myocardial infarction no benefit could be detected in the treatment group (Hausenloy et al., 2014; Cung et al., 2015). One possible explanation for the failure of cyclosporine A was that the drug did not reach the infarcted area in sufficient concentrations due to coronary occlusion.

Although it is known that cardioplegia induces AIF and cC3 translocation (Ramlawi et al., 2006), both apoptotic markers were low in our cardiac specimen after cardioplegia and also were not different between the experimental groups. An explanation for this phenomenon could be that the time frame of 60 min reperfusion after cardioplegia was too short to detect apoptotic or necrotic cells. Though, the interval from beginning of an apoptotic stimulus until AIF or cC3 nuclear translocation might be tissue dependent and also dependent on the kind of apoptotic stimulus it will normally take several hours until apoptosis is complete (Schmitt et al., 2002; Meggyeshazi et al., 2014).

As mentioned above, ATP levels were higher in the olesoxime treated hearts, thus, if olesoxime has the capacity to maintain ATP-synthesis via blockade of prolonged MPTP-opening it should improve the metabolic state of cells and also should ameliorate cardiac function after cardioplegia. Indeed, we could detect an enhanced contractility and improved relaxation behavior of the left ventricle in the CP+ group. In conclusion, it can be said that olesoxime application during cardioplegia improved cardiac outcome and thus might be a therapeutic tool during this condition.

Taken together, our data show that in isolated rabbit hearts cardioplegia results in delayed recovery of contractility and lusitropy together with reduced ATP content and slowed ventricular conduction, so that the hearts became more prone to ventricular fibrillation. These effects of cardioplegic arrest and the resulting functional defects could be significantly alleviated by olesoxime. Our findings that olesoxime treatment in addition to these effects also decreased PAR-synthesis, HSP60 expression and nitrotyrosine formation are in good accordance with the hypothesis, that the blockade of MPTP might be the underlying effect for the beneficial actions of olesoxime.

The results of the present study -referring to ischemia-related changes- may encourage further studies targeted at local infarction and infarct size reduction.

### Limitations of the study

As all methods the isolated Langendorff heart has also limitations. Among these we should be aware, that the heart is perfused with saline solution and not with blood. Thus, aspects of O_2_ delivery and aspects regarding inflammatory responses can differ from the *in vivo* situation. Another aspect concerns the EDP. In cardiac surgery typically the heart is emptied during arrest and refilled at reperfusion. Due to the fact that the 256 electrodes had to stay in place we could not unload the balloon, repress and refill the balloon for technical reasons, since otherwise the geometry of the ventricle would be changed and the electrodes would alter their location by these manual manipulations.

Although desirable we had no possibility to perform electron microscopy or to measure mitochondrial function and ROS production directly. However, we measured NT production as a surrogate parameter for nitrosative stress. Thus, the conclusions on an involvement of mitochondria are indirect at present and need further validation in subsequent studies targeted directly at mitochondrial function.

## Author contributions

AS, ID, and SD conceived and designed the experiments for this manuscript. AS, MK, and SD performed the experiments, analyzed and interpreted the data of this work. AS and SD wrote the manuscript.

### Conflict of interest statement

The authors declare that the research was conducted in the absence of any commercial or financial relationships that could be construed as a potential conflict of interest.
